# Challenges in Transition From Childhood to Adulthood Care in Rare Metabolic Diseases: Results From the First Multi-Center European Survey

**DOI:** 10.3389/fmed.2021.652358

**Published:** 2021-02-25

**Authors:** Karolina M. Stepien, Beata Kieć-Wilk, Christina Lampe, Trine Tangeraas, Graziella Cefalo, Nadia Belmatoug, Rita Francisco, Mireia del Toro, Leona Wagner, Anne-Grethe Lauridsen, Sylvia Sestini, Nathalie Weinhold, Andreas Hahn, Chiara Montanari, Valentina Rovelli, Cinzia M. Bellettato, Laura Paneghetti, Corine van Lingen, Maurizio Scarpa

**Affiliations:** ^1^Adult Inherited Metabolic Diseases, Salford Royal NHS Foundation Trust, Salford, United Kingdom; ^2^Department of Metabolic Diseases and Diabetes, Krakow University Hospital, Krakow, Poland; ^3^Department of Metabolic Diseases, Medical College, Jagiellonian University, Krakow, Poland; ^4^Department of Child Neurology, Center for Rare Diseases Giessen (ZSEGI), Justus-Liebig University, Giessen, Germany; ^5^Norwegian National Unit for Newborn Screening, Division of Paediatric and Adolescent Medicine, Oslo University Hospital, Oslo, Norway; ^6^Department of Maternal and Child Health, San Paolo Hospital, University of Milan, ASST Santi Paolo e Carlo, Milan, Italy; ^7^Referral Center for Lysosomal Diseases, AP-HP Nord, Beaujon Hospital, Paris University, Clichy, France; ^8^Portuguese Association for Congenital Disorders of Glycosylation and other Rare Metabolic Diseases, Lisbon, Portugal; ^9^Pediatric Neurology Department, University Hospital Vall d'Hebron, Universitat Autónoma de Barcelona, Barcelona, Spain; ^10^German-Speaking Self-Help Group for Alkaptonuria (DSAKU) e.V., Stuttgart, Germany; ^11^International Gaucher Alliance, Dursley, United Kingdom; ^12^Gaucher Association Denmark, Holbaek, Denmark; ^13^Italian Association of Patients With Alkaptonuria (aimAKU), Siena, Italy; ^14^Metabolic Unit, Interdisciplinary Centre for Metabolism: Endocrinology, Diabetes and Metabolism (UP) and Children's Hospital, Charité University Hospital Berlin, Berlin, Germany; ^15^Department of Child Neurology, Justus-Liebig University, Giessen, Germany; ^16^MetabERN, Regional Coordinating Center for Rare Diseases, Udine University Hospital, Udine, Italy

**Keywords:** rare disease, inherited metabolic disease(s), transition process, challenge, adulthood (18 years and older), continuity of care, adult metabolic patient

## Abstract

Inherited Metabolic Diseases (IMDs) are rare diseases caused by genetic defects in biochemical pathways. Earlier diagnosis and advances in treatment have improved the life expectancy of IMD patients over the last decades, with the majority of patients now surviving beyond the age of 20. This has created a new challenge: as they grow up, the care of IMD patients' needs to be transferred from metabolic pediatricians to metabolic physicians specialized in treating adults, through a process called “transition.” The purpose of this study was to assess how this transition is managed in Europe: a survey was sent to all 77 centers of the European Reference Network for Hereditary Metabolic Disorders (MetabERN) to collect information and to identify unmet needs regarding the transition process. Data was collected from 63/77 (81%) healthcare providers (HCPs) from 20 EU countries. Responders were mostly metabolic pediatricians; of these, only ~40% have received appropriate training in health issues of adolescent metabolic patients. In most centers (~67%) there is no designated transition coordinator. About 50% of centers provide a written individualized transition protocol, which is standardized in just ~20% of cases. In 77% of centers, pediatricians share a medical summary, transition letter and emergency plan with the adult team and the patient. According to our responders, 11% of patients remain under pediatric care throughout their life. The main challenges identified by HCPs in managing transition are lack of time and shortage of adult metabolic physician positions, while the implementations that are most required for a successful transition include: medical staff dedicated to transition, a transition coordinator, and specific metabolic training for adult physicians. Our study shows that the transition process of IMD patients in Europe is far from standardized and in most cases is inadequate or non-existent. A transition coordinator to facilitate collaboration between the pediatric and adult healthcare teams should be central to any transition program. Standardized operating procedures, together with adequate financial resources and specific training for adult physicians focused on IMDs are the key aspects that must be improved in the rare metabolic field to establish successful transition processes in Europe.

## Introduction

Inherited Metabolic Diseases (IMDs) encompass an expanding and comprehensive group of rare diseases caused by inherited defects in various biochemical pathways. Currently, IMDs include more than 1,400 different genetic diseases (1) that can be classified into 130 biochemical groups according to the underlying metabolic pathway (2). The first symptoms of IMDs are often non-specific and overlap with more common disorders, which delays diagnosis and frequently results in organ dysfunction or failure. Although the individual incidence is low (from 1 in 10,000 to 1 in 1 million), the cumulative incidence of all IMDs is high, ranging from 1 in 800 to 1 in 2,500 new-borns (3–8).

Clinical presentation and course of IMDs are variable, spanning from acute life-threatening metabolic decompensation in the new-born period (e.g., hyperammonemic encephalopathy) to a slowly progressive disease with initial symptoms manifesting only in adulthood (9). Given the progressive nature of IMDs, early diagnosis and treatment initiation are extremely important, as this can slow down or even halt the progression of the disease. In addition, because of the variability and complexity of IMDs, highly specialized, experienced, and coordinated multidisciplinary teams are required in order to minimize negative health effects and to sustain patients' quality of life.

Earlier diagnosis and advances in treatment have much improved the prognosis and life expectancy of IMDs over the last decades, meaning that more than 90% of rare metabolic patients will survive beyond the age of 20 years (10, 11). Moreover, the expanded use of next generation sequencing both widens the phenotypic spectrum within known diseases and reveals new IMDs. However, the increased survival has created a number of new issues and challenges: the development of long-term age-related complications, the metabolic progression of the underlying condition, and the lack of data on the natural course of the disease. These new challenges require the care of adolescent IMD patients being transferred from metabolic pediatricians to metabolic physicians specialized in treating adults to an increasing extent, including the development and coordination of a multidisciplinary team for each individual IMD (12). An adult metabolic team is defined as a core multidisciplinary group consisting of the following health professions: adult physician, specialized nurse and/or patient coordinator, metabolic dietician, and with access to other sub-specialists.

The transition process is critical to ensure that adolescent patients with IMDs obtain the best quality of life possible as adults. Also, patients and families need to become empowered and take full responsibility of their disease. To this end, an appropriate and gradual transition program is pivotal: patients need to be informed and accompanied step-by-step as they gradually switch from a pediatric care in which doctors and caregivers are responsible for the patient—from organizing medical visits to buying medications—to an adult care in which the patient is aware of all the precautions and treatments to manage her/his condition (under control) and avoid deterioration. Only through a successful and gradual transition program will an IMD patient become fully independent and capable of taking life-long care of her/his health (13).

Transitional care has been defined as “the purposeful, planned movement of adolescents and young adults with chronic physical and medical conditions from child-centered to adult-oriented health care systems” (14). But up to now little has been done to ensure that transition in IMDs is performed in a formalized, standardized, and authoritative manner. Although current literature on transition for chronic diseases in general is quite extensive (15–17), for IMDs only a few centers have created specific transition guidelines (12, 18, 19). In addition, an overview of the different transition practices and challenges in Europe has never been attempted. The collection of these important data can serve as a starting point for common European transition best practice recommendations.

The European Reference Network for Hereditary Metabolic Disorders (MetabERN), established in 2017, connects centers specialized in rare metabolic diseases at EU level; it represents 77 healthcare providers (HCPs) from 23 EU Member States and 44 patient organizations. It is also endorsed by the Society for the Inborn Errors of Metabolism (SSIEM). Overall, the network follows almost 33,000 IMD patients (16,586 adults and 16,277 pediatric patients as of November 2020). MetabERN is organized in nine work packages (WP) and in seven subnetworks (SNW), each SNW being specific for the metabolic defects and/or pathways involved in the disease (for details please refer to https://metab.ern-net.eu). The WP9, representing patient empowerment, has highlighted the need to take urgent care of the transition process. In 2019, MetabERN and SSIEM organized a webinar in which four MetabERN centers presented their own program on transition. Then, in the context of the WP4 on Guidelines, Care Pathways and Standardization for Medical Care and Transition, MetabERN established the Transition Project Working Group (TPWG), which is led by referral experts in the field in collaboration with the associations of patients.

As a first step in its activities, the TPWG has investigated how the transition process is currently organized in European metabolic centers, in particular what transition programs currently exist, whether there is any reimbursement for metabolic patients available, and how the transition process could be supported further to facilitate smooth transition for metabolic patients.

For this purpose, a survey was created and sent to all the MetabERN centers to collect information and to identify unmet needs. The survey covered not only medical, but also organizational, structural, social, administrative and educational issues in order to explore and assess potential difficulties in the organization of an efficient transition process from the point of view of physicians dealing with patients with IMDs. This initial overview of the current status of transition in Europe is essential to raise awareness on the issue at a national and European level.

## Materials and Methods

The Survey Monkey platform was used to design the survey and collect the data. Invitations with the link to access the platform were sent via email to all 77 MetabERN centers in 23 EU countries. The survey included 34 questions aimed at gathering information on the status of the transition process, its organization and the associated difficulties and needs from the perspective of HCPs (see Supplementary Material for full list of questions). The survey included multiple choice questions, with the possibility to write additional text under the option “Other” when available; only in the last question participants were asked to add any relevant comment as free text (see Supplemental Material). Members of the TPWG prepared the survey in collaboration with adult IMD patients and patients associations (see section Acknowledgments). The survey was active for 20 months, from 1st October 2018 to 1st May 2020. All participants (see section Acknowledgments) gave their consent for data collection and publication. Data was extracted and descriptive statistical analysis was performed using Microsoft Excel.

## Results

Data was collected from 63/77 (81%) HCPs from 20 EU countries (Table 1). Responders were mostly metabolic pediatricians (65.1% pediatric vs. 11.1% adult metabolic physicians; 23.8% clinical geneticists and other specialties), with representatives covering all disease SNW, but dominated by the largest disease entities: lysosomal storage disorders (LSD; 87.1%), amino and organic acids related disorders (AOA; 83.9%), and carbohydrate, fatty acid oxidation and ketone bodies disorders (C-FAO; 80.7%) (Table 1).

**Table 1:** Characteristics of the responders and the relative centers.

**Category**	***n* (%)**
**Profession:**	
Metabolic pediatrician	41/63 (65.1)
Adult metabolic physician	7/63 (11.1)
Clinical geneticist	3/63 (4.8)
Other *(neurologist, cardiologist, psychologist, genetic counselor, clinical biochemist, internal medicine/endocrinology/diabetes specialist)*	12/63 (19)
**Metabolic conditions followed by the responder:**	
LSD	54/63 (87.1)
AOA	52/63 (83.9)
C-FAO	50/63 (80.7)
PM-MD	44/63 (71)
CDG	38/63 (61.3)
PD	37/63 (59.7)
NOMS	37/63 (59.7)
**Center status:**	
Adult and pediatric center	53/63 (84.1)
Pediatric center only	6/63 (9.5)
Adult center only	4/63 (6.4)
**Center following adult patients with IMDs:**	
Yes	59/63 (93.7)
No	4/63 (6.4)
**Center with separate adult metabolic team:**	
Yes, for all kinds of metabolic conditions	19/62 (30.7)
Yes, for the majority of metabolic conditions	12/62 (19.4)
Yes, for selected groups of metabolic conditions	12/62 (19.4)
No, pediatric team follows patients life-long	5/62 (8.1)
**Reasons for not having an adult metabolic team:**	
Lack of interest in IMDs among adult physicians	9/26 (34.6)
Patient/caregiver's preference to be followed by pediatric metabolic department	8/26 (30.8)
Lack of special training for adult physicians in metabolic diseases in the country	8/26 (30.8)
No existing position/vacancy for adult metabolic diseases at the center	8/26 (30.8)
Lack of financial support	5/26 (19.2)
Lack of extra reimbursement for adult complex metabolic patients	5/26 (19.2)
Lack of adult physicians willing to be involved	4/26 (15.4)
Other *(historically, adult IMD patients have been taken care in the pediatric hospital; difficulties in getting more salaries for adult physicians; smooth transition by joint follow-up with both adult and pediatric physician until the patient agrees to be followed by the internist)*	14/26 (53.9)
**Age at which transition process starts:**	
18 years of age	33/63 (52.4)
16 years of age	12/63 (19)
20 years of age	2/63 (3.2)
Other *(at 10 or 14 years of age; after 18 years of age or later; from 16 to 28 years of age, start discussion on transition from age 12; no transition process)*	16/63 (25.4)
**Age at which transition process is finalized:**	
>18 years of age	32/56 (57.1)
16–18 years of age	12/56 (21.4)
Never, because the patient remains under pediatric care throughout his/her life	6/56 (10.7)
14–16 years of age	0/56 (0)
12–14 years of age	0/56 (0)
Never, because the patient is transferred to an adult clinic without any preparation	0/56 (0)
Other *(for older patients the transition is finalizing now, regardless of age, while for Fabry and other patients it is finalized at 16 years of age; depends on the disease: some patients remain at least in part managed by pediatricians)*	6/56 (10.7)

*AOA, Amino and organic acids-related disorders; PM-MD, Disorder of pyruvate metabolism, Krebs cycle defects, mitochondrial oxidative phosphorylation disorders, disorders of thiamine transport and metabolism; C-FAO, carbohydrate, fatty acid oxidation and ketone bodies disorders; LSD, lysosomal storage disorders; PD, peroxisomal disorders; CDG, congenital disorders of glycosylation and disorders of intracellular trafficking; NOMS, disorders of neuromodulators and other small molecules*.

Of the responding centers, 6.4% are dedicated exclusively to adults, 84.1% of centers follow both adult and pediatric patients, while 9.5% are pediatric only (Table 1). Overall, almost all responding centers follow adult patients (93.7%), and the majority of these have a separate adult metabolic team. However, in only 30.7% of cases is the adult metabolic team available for all metabolic conditions (Table 1). The main reasons identified for the centers not to have an adult team include: (i) no interest in the metabolic field from adult physicians (34.7%); (ii) no existing position (30.8%); (iii) lack of specialty adult training in the country (30.8%); and (iv) preference of patient/family to be followed by the pediatric team (30.8%) (Table 1).

In centers with existing transition programs, for over half the cases (52.4%) transition starts at 18 years of age. Consequently, the process is mostly finalized after the age of 18 (57.1 vs. 21.4% between 16 and 18 years of age), while 10.7% of patients never transition because they remain under pediatric care throughout their life (Table 1). Of the doctors involved in the transition process, the majority of the respondents (87.7%) discuss the transition issue with the adolescent patients and parents, but <50% have a separate consultation with the child/adolescent.

Medical specialities involved in the medical care of adult metabolic patients are mainly internists (50%), followed by clinical geneticists and cardiologists (15.6% each) (Table 2). In the majority of centers (80.7%) the adult patients are followed also by other specialists, mainly neurologists (77.8%), cardiologists (64.8%), nephrologists (61.1%), and nutritionists (59.3%) (Table 2). Importantly, it should be noted that among cardiologists and nephrologists participating in the survey, also pediatricians were represented, further increasing the percentage of pediatricians caring for adult patients with an IMD. In most centers there is no designated transition coordinator (69.5%) and no dedicated physician in charge of the transition on the adult side that collects and summarizes information from each sub-specialist (62.1%) (Table 2).

**Table 2:** Specialties involved in the care of adult IMD patients and their transition.

**Category**	***n* (%)**
**Specialty of the physician taking care of adult IMD patients:**	
Internist	16/32 (50)
Clinical geneticist	5/32 (15.6)
Cardiologist	5/32 (15.6)
Neurologist	4/32 (12.5)
Diagnosis, geneticist	2/32 (6.3)
Dietician/nutritionist	2/32 (6.3)
Orthopedist	2/32 (6.3)
Ophthalmologist	2/32 (6.3)
Radiologist	2/32 (6.3)
Social worker	2/32 (6.3)
Surgeon	1/32 (3.1)
Clinical trial technician	1/32 (3.1)
Coordinator/secretary	1/32 (3.1)
ICU and anesthetist	1/32 (3.1)
Gastroenterologist	1/32 (3.1)
Genetic counselor	1/32 (3.1)
Medical biochemist	1/32 (3.1)
Neuro-pediatrician	1/32 (3.1)
Otorhynolaryngologist	1/32 (3.1)
Palliative care	1/32 (3.1)
Pharmacist	1/32 (3.1)
Physical and rehabilitation medicine	1/32 (3.1)
Psychologist	1/32 (3.1)
Pneumologist	1/32 (3.1)
Special nurses	1/32 (3.1)
Epidemiologist	0/32 (0)
Internist-rheumatologist	0/32 (0)
Hepatologist	0/32 (0)
Neuropathologist	0/32 (0)
Neuropsychologist	0/32 (0)
Psychiatrist	0/32 (0)
School educator	0/32 (0)
Stomatologist	0/32 (0)
Other *(endocrinologist, nephrologist, pediatrician, metabolic physician)*	8/32 (25)
**Other specialists involved in the care of adult IMD patients:**	
Neurologist	42/54 (77.8)
Cardiologist	35/54 (64.8)
Nephrologist	33/54 (61.1)
Nutritionist	32/54 (59.3)
Clinical geneticist	22/54 (40.7)
Ophthalmologist	21/54 (38.9)
Hepatologist	20/54 (37)
Hematologist	19/54 (35.2)
Endocrinologist	19/54 (35.2)
Laboratory geneticist	17/54 (31.5)
Rehabilitation specialist	14/54 (25.9)
Gastroenterologist	12/54 (22.2)
General internal medicine	12/54 (22.2)
Rheumatologist	9/54 (16.7)
Psychiatrist	8/54 (14.8)
**Designated transition coordinator at the center:**	
Yes	18/59 (30.5)
No	41/59 (69.5)
**Dedicated physician collecting information from each specialist at the center:**	
Yes	22/58 (37.9)
No	36/58 (62.1)

Almost half of the centers (48.2%) provide a written individualized transition protocol, which is standardized in nearly 20% of cases (Table 3). In the majority of centers (63.4%) no patient organization has been involved in the development of the transition plan (Table 3). In almost 77% of centers pediatricians share a medical summary, transition letter and an emergency plan (if applicable) with the adult team and the patient (Table 3). This document contains information such as medications and relative dosage, comorbidities, a short summary of the disease and precautions, an emergency regime, a nutrition plan when healthy, and the last blood test results (Table 3).

**Table 3:** Information exchanged among physicians for/during transition.

**Category**	***n* (%)**
**Written individualized plan/protocol/letter for transition provided:**	
No, no particular documents are provided other than the medical record itself	22/56 (39.3)
Yes, a written individualized document for transition is provided, but it is not standardized	15/56 (26.8)
Yes, the hospital has a standard operating procedure for transition	6/56 (10.7)
Yes, a written standardized individualized protocol for each patient is provided	5/56 (8.9)
Yes, a document for transition is provided, but without knowing which physician to address it to if the patient will be followed at another hospital	1/56 (1.8)
No, but an unwritten transition agreement/procedure is arranged with the patient	1/56 (1.8)
Other *(ongoing protocol setup, summary letter, full documentation)*	6/56 (10.7)
**Relevant patient organization(s) involved in the development of a standard procedure for transition, where this is available:**	
Yes	8/41 (19.5)
No	26/41 (63.4)
Other	7/41 (17.1)
**Pediatric team sharing medical summary, transition passport or letter and/or emergency care plan with adult team and patient:**	
Yes	43/56 (76.8)
No	8/56 (14.3)
Other *(emergency plan only, hospital discharge summary, in the making)*	5/56 (8.9)
**Information included in the medical summary:**	
Medications	45/49 (91.8)
Short summary of disease and precautions	44/49 (89.8)
Updated list of medications and dosages	44/49 (89.8)
Comorbidities	42/49 (85.7)
Detailed emergency regime	39/49 (79.6)
Last blood test results	38/49 (77.6)
Nutritional plan when healthy	36/49 (73.5)
Medications to avoid	29/49 (59.2)
Suggested blood tests when admitted to hospital	28/49 (57.1)
Anesthesia precautions	26/49 (53.1)
Surgery preparation instructions	24/49 (49)
Other *(pregnancy precautions, recurrence risk, emergency regime, full history radiological images, psychological tests report, special needs)*	13/49 (26.5)

Among the responding physicians, only 39.7% have received appropriate training in managing health issues in adolescent metabolic patients, while the others acquired the necessary knowledge mainly through courses and internships in metabolic centers (Table 4).

**Table 4:** Shortcomings, challenges and needs related to transition.

**Category**	***n* (%)**
**Members of team looking after adolescent patients with formal training in health issues in adolescent IMD patients:**	
Yes	23/58 (39.7)
No	31/58 (53.5)
Other *(SSIEM courses and conferences, only for some IMD, only for general health issues in adolescents)*	4/58 (6.9)
**Informal methods used to acquire knowledge on treating adolescent IMD patients:**	
Course	13/36 (36.1)
Placement in metabolic center	13/36 (36.1)
Non-recognized education program	11/36 (30.6)
Fellowship	9/36 (25)
Other *(personal study, clinical practice, part of another specialization, prolonged work with IEM, cooperative interaction)*	12 (33.3)
**Most difficult challenges in managing the transition process:**	
Lack of time	29/59 (49.2)
Lack of adult metabolic physician positions/vacancies	26/59 (44.1)
Knowledge gaps on the topic amongst medical staff	17/59 (28.9)
Lack of reimbursement	14/59 (23.8)
Lack of interest	9/59 (15.3)
Poor communication between pediatric and adult centers	7/59 (11.9)
Other *(lack of pediatric metabolic physicians, growing number of patients, lack of cases manager, metabolic medicine is not available as specialization, no financial support, no official metabolic position, few doctors dedicated, low number of pts/condition and diverse patient population)*	17/59 (28.8)
**Most needed implementations for a successful transition:**	
More staff specifically dedicated to transition	31/60 (51.7)
A dedicated transition coordinator	27/60 (45)
Special metabolic training for adult physicians	26/60 (43.3)
Dedicated (or interested) adult physicians	21/60 (35)
Adult metabolic positions	19/60 (31.7)
A transition protocol/standard operating procedure	19/60 (31.7)
A physical space for transition clinics	17/60 28.3)
Other *(setup of a transition clinic; adult metabolic clinic; support by paramedics such as dietitians, psychologists, social workers; more protocols for adult patients; more pediatricians and metabolic adult physicians; more time to dedicate to transition)*	8/60 (13.3)
**Availability of additional financial support for transition clinics:**	
Yes	3/60 (5)
No	54/60 (90)
Other *(information unknown; no transition in place, process for improvement is ongoing)*	3/60 (5)

The main challenges of HCPs in managing the transition process are lack of time (49.2%) and shortage of adult metabolic physician positions (44.1%) (Table 4). This is reflected in the implementations that, according to the responders, are most required for a successful transition: medical staff dedicated to transition (51.7%), a transition coordinator (45%), and specific metabolic training for adult physicians (43.3%) (Table 4). Lastly, 90% of responders report the absence of any financial support for transition programs (Table 4).

## Discussion

This survey provides the first European report on the status of the transition process in rare metabolic diseases from the HCPs perspective. With 63 centers responding from 20 EU countries, our results may provide a representative overview of the current situation in European centers of excellence, selected for their expertise in metabolic diseases. The pediatric specialty was the most prevalent responder group in our survey, reflecting predominantly the pediatricians' point of view in the transition process. At the same time, our surveys show a large disproportion between the medical specialties looking after IMD patients. This underlines the multisystemic nature of IMDs and the peculiar need of affected patients to be followed by multiple professionals, who should interact and collaborate with one another within a well-defined multisystemic approach framework. Unfortunately, in a real world situation patients are seen by different specialists that rarely know or communicate with each other, resulting in a fragmented, uncoordinated and suboptimal care.

Here we show that most HCPs discuss transition with the family and share a medical summary with the adult physician and the patient. In most cases, this summary is not standardized and includes a short description of the disease, its precautions and comorbidities, type and dosage of medications, a detailed emergency regime, a nutrition plan, and the latest blood test results. Despite decades of increasing knowledge regarding the importance of a properly structured transition for later health outcomes (20, 21), our survey still demonstrates unmet needs and overall slow acquisition of the mandatory aspects for a successful transition for rare metabolic patients, their families and adult physicians in Europe.

In most centers a transition coordinator is missing and the process is fragmented or even non-existent, to a point where about 10% of patients never transition and stay under pediatric care all their life. In this context, the presence of a transition coordinator is a major factor for a successful transition program, as it ensures that adult care teams are aware of, and prepared for, the management of rare conditions and their peculiarities. The coordinator schedules the transition meetings, collects and updates all the necessary documentations, and ensures that all appropriate specialists and social professionals are present during the transition visits. Despite these differences, the transition coordinator should have defined duties to ensure the highest standards and success of the transition process. Our survey identified that the appointment of a transition coordinator is necessary, which calls for immediate action from healthcare organizations and policy makers to improve the transition process across Europe. The need for this new administrative role has also been identified by De Castro et al. (22).

Another main finding in our study is the shortage of physicians specialized in the adult care of IMDs. In fact, only 11% of the respondents to the survey were adult IMD professionals and most centers stated that an adult team was only available for a subset of metabolic diseases. In this context, the lack of specific metabolic training for adult physicians regarding adolescent health issues must be emphasized, as this is a key factor for implementing a successful transition program. Education of adult specialists in IMDs is important for a number of reasons: not only do an increasing proportion of pediatric patients survive with more complex disorders and with neurocognitive disabilities, but a greater number of IMDs presenting in adulthood are being diagnosed in the genomic era. As a part of education and optimization of the transition process, it is important to develop cooperation and trust between pediatricians and adult physicians (22). This will facilitate a more harmonious and less stressful transition process for the IMD patients.

The SSIEM recently launched a survey to 89 adult specialist members. Despite the fact that practical clinical experience with adult IMDs was considered key for their own education in rare metabolic diseases, most responders (73%) judged their education as poor or fair. The main message was the need for formal training opportunities in adults and courses on IMDs (23). These results were confirmed by our survey, in which only about 40% of responders reported formal training in health issues regarding adolescent IMD patients, while informal training such as fellowships, short courses, work placement, clinical practice and personal studies were common. As a result, neither pediatric nor adult clinicians are prepared to adequately address the complex developmental challenges that characterize adolescent metabolic patients. Indeed, our survey indicates that the factors that are mostly needed by HCPs are: adult physician positions, a transition coordinator, and specific metabolic training for adult physicians. The shortage of adult metabolic specialists has increasingly been revealed by the advancements made over the last few decades in metabolic diseases. Adult physicians have historically not been involved in the management of IMDs because until about 20 years ago 75% of metabolic patients died before reaching the adult age, resulting in a “skewed population of providers” (22). Today, thanks to better healthcare, improved follow-up programs, and more treatments available there is an increasing number of metabolic patients that reach adulthood. In this regard, both SSIEM and MetabERN are already organizing dedicated courses to create and train a new class of professionals, and in particular adult physicians, with expert knowledge and practical experience in the long-term care and management of IMDs. However, additional support is needed from hospital managements, authorities, and the European Commission to encourage adult physicians to focus on IMD patients. This will be important also to overcome another aspect highlighted by our survey, that is the lack of interest in IMDs by medical students and/or adult physicians. As rare and complex diseases, IMDs are not well known and are often overlooked in the medical community; therefore, more effort is needed to disseminate information about the challenges and opportunities offered by the metabolic disease field and thus create novel interest in this specialty.

Our results show that adult IMD patients are regularly followed mainly by internists, with the widespread involvement of other sub-specialists, illustrating the complexity of the diseases. This is not surprising, as most adults with a chronic condition demand surveillance by different specialists; however, this also highlights the fact that healthcare is more fragmented in adulthood. This might create obstacles in guaranteeing a continuous and holistic care of adult metabolic patients. Therefore, it is necessary to create a multidisciplinary team of HCP specialized in the treatment of adult patients with IMDs; for example, a team coordinated by an internist specialist in metabolic diseases. Regarding the type of specialties following IMD patients, these include mostly neurologists, cardiologists, nephrologists, orthopedist and nutritionists. This is not surprising, as it is a reflection of the multi-organ involvement of IMDs and the importance of a balanced diet to avoid metabolic decompensation.

The whole process of transition is further complicated by the lack of standardized programs or specific guidelines shared across Europe. This is a crucial aspect that needs to be addressed by institutions and policy makers in order to ensure that the best possible care—and therefore quality of life—is given not only to IMD and other rare disease patients, but to all patients with a chronic condition that arises during childhood. Indeed, the transition issues that were highlighted with our survey may be shared by other chronic conditions. As a possible solution strategy, the optimization of electronic medical records could be of help.

The lack of financial resources is another aspect that makes it difficult to implement appropriate transition teams and programs. In the midst of the current COVID-19 pandemic, which imposed great changes in healthcare funds and organization, rare diseases must not be forgotten and specific activities should be put into place to increase awareness in public institutions and authorities about IMDs and the transition process.

From the patient perspective, there are significant challenges to be considered when transitioning from a family-centered, developmentally-focused, and multidisciplinary pediatric care to a less supportive adult healthcare system that is often unfamiliar with rare diseases (13). Among them, there is the resistance and lack of trust of the patient and his/her family in regard to the adult team, and the fear of a lack of expertise in the adult specialists. Indeed, in a recent study on expectations of adolescents with chronic disorders and their parents, the most important barriers identified for successful transition were anxiety and lack of information of the adult healthcare specialist (24). Therefore, it is important to predispose combined consultations with the pediatric and adult specialists, to anticipate the exchange of medical records, and to establish clear communication strategies, which can be extremely beneficial in mitigating these difficulties and in making the patient and the caregivers feel more comfortable and continually cared for at the highest standards. Better information and education on their disease, as well as greater active involvement in the decision-making of their care, may be pivotal to improve patient and family adherence to and satisfaction with transitioning (22, 25). In this context, the role of patient associations is important to promote such measures. In addition, from a patient point of view, the transition process is multidimensional, involving transitions with respect to development (adolescent becoming a young adult), situation (switch from pediatric services to adult health services) and health–illness (role changes, self-management of a chronic condition) (25). Due to the heterogeneity of IMDs regarding organ involvement and disease severity, an interdisciplinary framework of care should be introduced stepwise to meet the biopsychosocial needs of early adolescents (11–15 years of age), late adolescents (16–18 years old), and emerging adults (18–25 years of age), thereby also differentiating between life-threating IMDs, IMDs with chronic illness, and IMDs with severe disabilities and/or severe intellectual impairments (11, 25, 26). Since the current survey did not target patients and their families, a dedicated survey is necessary to focus on their point of view.

This study has several limitations. Firstly, it provides only the MetabERN HCPs' point of view. More specifically, the majority of respondents were pediatricians, as they have been the historical managers of patients with IMD, leaving the adult perspective of the transition process less represented. Moreover, transition is a process involving several key players in the center of interest; therefore, the next survey to complement that of the HCPs should be targeted at patients and their associations to understand their point of view and especially to gather information on the social and psychological aspects of transition, which seems even more difficult in patients with multiple disabilities. Our survey focused on HCPs, which can provide information and suggestions regarding the more practical and administrative aspects of the transition process. In fact, no publications exist on the patient or caretaker point of view and indeed this is one of the next steps to be taken by MetabERN. Secondly, due to the design of the survey, we were not able to collect specific data regarding the percentage of pediatricians that are involved in the care of adult IMD patients. The majority of our responders (65%) were pediatricians and at present we are not able to assess whether other specialists taking care of adult patients are also pediatricians. Thirdly, specific questions about the set-up and maintenance of a successful transition process were missing, so at this stage we cannot provide exhaustive examples or recommendations on how to improve the transition where this is difficult. Further work is needed to propose specific transition recommendations in the field of IMDs, for example by using templates from hospitals that have a long experience with transition and have made some written recommendations, also taking into account the patients' perspective. Indeed, this is part of the further activities planned by MetabERN's TPWG.

The final aim of MetabERN and the TPWG is to collaborate and share expertise and good practices to develop possible action guidelines and minimal standard of care. In this way, all specialists involved in the care of IMD patients, and potentially in other fields, will have access to guidance and support in the management of the critical process of transition, which in turn will aid a successful and efficient transfer from pediatric to adult care for all patients. This study is the first step in that direction.

## Data Availability Statement

The datasets presented in this article are not readily available because they are stored in a private depository. Requests to access the datasets should be directed to Maurizio Scarpa, maurizio.scarpa@metab.ern-net.eu.

## Ethics Statement

Ethical review and approval was not required for the study on human participants in accordance with the local legislation and institutional requirements. The patients/participants provided their written informed consent to participate in this study.

## Author Contributions

GC, A-GL, and KMS developed the survey and revised the manuscript. NB, MT, RF, AH, BK-W, CL, CM, TT, VR, SS, KMS, LW, NW, and CvL revised the manuscript. CMB and LP analyzed the data and wrote the manuscript. MS supervised the study and revised the manuscript. All authors contributed to the article and approved the submitted version.

## Conflict of Interest

The authors declare that the research was conducted in the absence of any commercial or financial relationships that could be construed as a potential conflict of interest.

## Abbreviations

AOA: amino and organic acids related disorders
C-FAO: carbohydrate, fatty acid oxidation and ketone bodies disorders
HCP: healthcare provider
IMD: inherited metabolic disease
TPWG: transition project working group
SNW: subnetwork
WP: work package.
