# Supplementary material for: Challenges in Transition From Childhood to Adulthood Care in Rare Metabolic Diseases: Results From the First Multi-Center European Survey
Source: Front Med (Lausanne). 2021 Feb 25;8:652358. doi: 10.3389/fmed.2021.652358 (PMC7962750; doi:10.3389/fmed.2021.652358)
Supplement: Supplementary file 1 [file Data_Sheet_1.PDF]

Transition Survey

1. Introduction

With this survey we invite you to give your input about the Transition process from paediatrics to adulthood (when, where, how) at your Centre.

This survey is part of the activity of the MetabERN Work Package 4 on Guidelines, Care Pathways and Standardization for Medical care and transition, which is a key component of MetabERN. This survey contains 34 questions and will take you about 18 minutes to fill in.

Transition is a very important and underestimated part of the care for metabolic patients and there is a lot of diversity in Europe and within countries on how to deal with transition. With this survey we are gathering important information and data on the state of transition, the way it is organised and the difficulties that are faced. MetabERN is planning to use the data that you will provide us, to publish and disseminate knowledge, raise awareness and start setting up plans and protocols on how to deal with transition for metabolic patients as well as the sharing of good practices.

The personal data collected from healthcare professionals (full name, organization, email address and country) will only be used for the purpose of the study, as determined by MetabERN, and will not be used or sold for any other purposes. Completed questionnaires, including personal data of the HCP, will be accessed and analysed by the Coordination Office of MetabERN conducting the survey and treated confidentially. The Coordination Office takes all appropriate technical and organizational measures in order to comply with the applicable data protection laws, including the General Data Protection Regulation (EU) 2016/679 as from 25 May 2018, and ensures the protection of the rights of healthcare professionals as data subjects. More information on the processing of personal data in the context of the MetabERN Survey can be found in the Privacy Policy of the Udine University Hospital that the Coordination Office is linked to. The questionnaire is also subject to SurveyMonkey's Terms of Use, including its Privacy Policy.

1. Do you agree to the above terms? By answering Yes, you acknowledge that you have read and understood our Privacy Policy and that you agree to take part in this questionnaire.

- ◯ Yes
- ◯ No

If your answer was yes, we kindly ask you to answer the questionnaire below and appreciate your valuable input in order to collect and share all relevant information regarding the Transition Process and how this is happening all over Europe. Please finish the whole surveyso that we will have a complete data set.

2. Before starting the questionnaire, please fill in your name, country, and mail address. If you want to remain anonymous, skip this part and go straight to the questionnaire although we urge you to at least provide us with the country that you are from; this is valuable information to us.

Name

Country

Organisation

Email address

3. Which metabolic conditions do you work with?

- ☐ Amino and organic acids-related disorders (AOA)
- ☐ Disorder of pyruvate metabolism, Krebs cycle defects, mitochondrial oxidative phosphorylation disorders, disorders of thiamine transport and metabolism (PM-MD)
- ☐ Carbohydrate, fatty acid oxidation and ketone bodies disorders (C-FAO)
- ☐ Lysosomal storage disorders (LSD)
- ☐ Peroxisomal disorders (PD)
- ☐ Congenital disorders of glycosylation and disorders of intracellular trafficking (CDG)
- ☐ Disorders of Neuromodulators and Other Small Molecules (NOMS)

Survey Questions

4. What is your profession?

- ○ Metabolic paediatrician
- ○ Adult metabolic physician
- ○ Clinical geneticist
- ○ Other (please specify)

5. What is the status of your centre?

- ○ An adult centre only
- ○ A paediatric centre only
- ○ Both

6. At what age does the transition process start in your Centre?

- ○ At 16 years of age
- ○ At 18 years of age
- ○ At 20 years of age
- ○ Other (please specify)

7. Does your centre follow adult patients affected by inborn errors of metabolism (IEM)?

- ◯ Yes
- ◯ No

8. If you answered yes in the previous question, do you have a separate adult metabolic team looking after adult patients?

- ◯ Yes, for all kinds of metabolic conditions
- ◯ Yes, for the majority of metabolic conditions
- ◯ Yes, but only for selected groups of metabolic conditions
- ◯ No, the paediatric team follows the patients life-long
- ◯ Other (please specify)

9. If you answered no in the previous question, what is the reason for not having established an adult metabolic team at your centre? (Tick the boxes that apply)

- ☐ The adolescents and his/her parent(s) prefer to continue to be followed-up at the paediatric metabolic department
- ☐ There is no financial support to establish a metabolic team
- ☐ There is not enough hospital management awareness to establish practical guidelines for the transition process, therefore there are no adult physicians willing to be involved
- ☐ There is no special training for adult physicians in metabolic diseases in my country
- ☐ Other (please specify)
- ☐ There is no interest in this field from adult physicians
- ☐ There is no extra reimbursement for adult complex metabolic patients
- ☐ There are no existing position/vacancy for adult metabolic diseases at my centre

10. Do other specialist teams in your hospital follow adult metabolic patients?

- ◯ Yes
- ◯ No

11. If you answered Yes in the previous question, please choose which specialities are involved. More than one option is possible; you can use a new row for more options.

	Specialities names	Are they part of the 'core' metabolic team?
Which of the listed specialists follow metabolic patients in your centre/hospital?		
Which of the listed specialists follow metabolic patients in your centre/hospital?		
Which of the listed specialists follow metabolic patients in your centre/hospital?		
Which of the listed specialists follow metabolic patients in your centre/hospital?		
Which of the listed specialists follow metabolic patients in your centre/hospital?		
Which of the listed specialists follow metabolic patients in your centre/hospital?		
Which of the listed specialists follow metabolic patients in your centre/hospital?		
Which of the listed specialists follow metabolic patients in your centre/hospital?		
Which of the listed specialists follow metabolic patients in your centre/hospital?		
Which of the listed specialists follow metabolic patients in your centre/hospital?		

12. Is there a dedicated physician in charge of the transition who collects the information from each specialist?

	If yes, what is the specialisation of the dedicated physician?
Yes	
No	

13. If you do have a transition clinic in your centre, where is it located?

- ◯ In the adult clinic
- ◯ In the paediatric clinic
- ◯ We do not have this in our centre

14. **To be filled by physician taking care of adult IEM patient only**:
Which specialty do you work in?

- ☐ Surgeon
- ☐ Clinical geneticist
- ☐ Cardiologist
- ☐ Clinical trial technician
- ☐ Coordinator/Secretary
- ☐ Diagnosis, geneticist
- ☐ Dietician/Nutritionist
- ☐ Epidemiologist
- ☐ ICU and anesthesist
- ☐ Internist
- ☐ Internist-Rhumatologist
- ☐ Gastroenterologist
- ☐ Genetic counsellor
- ☐ Hepatologist
- ☐ Medical biochemist
- ☐ Neuropaeditrician
- ☐ Neuropathologist
- ☐ Neurologist
- ☐ Neuropsychologist
- ☐ Otorhynolaryngologist
- ☐ Orthopedist
- ☐ Opthalmologist
- ☐ Palliative care
- ☐ Pharamcists
- ☐ Pharamcists
- ☐ Physical and Rehabilitation Medicine
- ☐ Psychiatrist
- ☐ Psychologist
- ☐ Pneumologist
- ☐ Radiologist
- ☐ School educator
- ☐ Social Worker
- ☐ Specialised nurses
- ☐ Stomatologist
- ☐ Other (please specify)

15. Which specialities are represented in the Transition team in your centre? I.e. who is present during the consultation in transition clinics?

- [ ] Surgeon
- [ ] Clinical geneticist
- [ ] Cardiologist
- [ ] Clinical trial technician
- [ ] Coordinator/Secretary
- [ ] Diagnosis, geneticist
- [ ] Dietician/Nutritionist
- [ ] Epidemiologist
- [ ] ICU and anesthesist
- [ ] Internist
- [ ] Internist-Rhumatologist
- [ ] Gastroenterologist
- [ ] Genetic counsellor
- [ ] Hepatologist
- [ ] Medical biochemist
- [ ] Neuropaeditrician
- [ ] Neuropathologist
- [ ] Neurologist
- [ ] Neuropsychologist
- [ ] Otorhynolaryngologist
- [ ] Orthopedist
- [ ] Opthalmologist
- [ ] Palliative care
- [ ] Pharamcists
- [ ] Pharamcists
- [ ] Physical and Rehabilitation Medicine
- [ ] Psychiatrist
- [ ] Psychologist
- [ ] Pneumologist
- [ ] Radiologist
- [ ] School educator
- [ ] Social Worker
- [ ] Specialised nurses
- [ ] Stomatologist
- [ ] Other (please specify)

16. Do you have a designated transition coordinator?

- ( ) Yes
- ( ) No

17. If you answered yes in the previous question, who is the designated person?

18. Does your centre/hospital provide a written individualized plan/protocol/letter for transition?

- ◯ Yes, my hospital has a standard operating procedure for transition (please share the concept with us by sending it to cinzia.bellettato@metab.ern-net.eu or corine.vanlingen@metab.ern-net.eu)
- ◯ Yes, we provide a written standardized individualized protocol for each patient
- ◯ Yes, we provide a written individualized document for the purpose of transition for each patients but it is not standardized
- ◯ Yes, we provide a document for transition but we do not know which physician to address it to if the patient will be followed at another hospital….
- ◯ No, we do not provide any particular documents other than the medical record itself
- ◯ Not at the Centre, but I use unwritten transition agreement/procedures with my patients
- ◯ Other (please specify)

19. If your centre has a standard procedure for transition, ha(s)(ve) relevant patient organization(s) been involved in the development?

- ◯ Yes
- ◯ No
- ◯ Other (please specify)

20. If you have a separate team for adult IEM patients, at what time do you start the transition process?

- ◯ At the last visit
- ◯ 6 months before transition
- ◯ 1 year before transition
- ◯ More than one year before transition
- ◯ Other (please specify)

21. How many transition consultations are usually scheduled for patients before they are ready to attend adult metabolic clinics?

- ◯ 1-2
- ◯ 3-4
- ◯ More than 5
- ◯ Depends on the maturity of the patient
- ◯ Other (please specify)

22. At what age do you usually finalize transition to the metabolic clinics for adults?

- 12-14 years
- 14-16 years
- 16-18 years
- >18 years
- Never, because patients remain under paediatric care throughout their life
- Never, because the patient is transferred to an adult clinic without special preparation.
- Other (please specify)

23. Does your health care team discuss transition with the adolescent patient and with the parents/carers?

- Yes
- No
- I don't know (please fill in the reason for this choice in the other/comment field)
- Other (please specify)

24. If you answered the previous question with Yes, when does the discussion with the patient and the parents take place?

- During the last visit
- 6 months before transition
- 1 year before transition
- Over one year before transition
- I don't know
- Other (please specify)

25. Does your health care team discuss transition with the child/adolescent patient alone?

- Yes
- No
- I don't know

26. At what age do you start having separate consultation with the child/adolescent regarding his /her metabolic condition?

- 10 years of age
- 11-12 years of age
- 12-14 years of age
- 14-16 years of age
- >16 years of age
- I seldom or never have separate consultations at any age

27. How would you describe the communication/relationship between paediatricians and the adult team at your centre?

- ◯ Very good
- ◯ Good
- ◯ Mediocre
- ◯ Poor

28. How would you describe the communication/relationship between paediatricians and the adult physicians if the patient has transitioned to a centre outside your centre of expertise?

- ◯ Very good
- ◯ Good
- ◯ Mediocre
- ◯ Poor
- ◯ Other (please specify)

29. If you choose mediocre or poor in the previous question: what is the reason for that?

- ☐ Lack of time
- ☐ Lack of interest
- ☐ We operate in different locations
- ☐ Adult physicians in charge are constantly replaced
- ☐ No common place to meet adult physicians
- ☐ Other (please specify)

30. Does the paediatric team share a current medical summary, transition passport or letter and/or emergency care plan with the adult team and patients?

- ◯ Yes
- ◯ No
- ◯ Other (please specify)

31. If you answered yes in the previous question; What does the medical summary contain?

- [ ] Short summary of the disease and precautions
- [ ] Medications,
- [ ] Comorbidities
- [ ] Nutritional plan when healthy
- [ ] Detailed emergency regime
- [ ] Medications to avoid
- [ ] Anaesthesia precautions
- [ ] Surgery preparation instructions
- [ ] Suggested blood tests when admitted to hospital
- [ ] Actual medication and dosages
- [ ] Last blood test results
- [ ] Other (please specify)

32. Do the members of the team looking after adolescent patients have any formal training in managing health issues in adolescent metabolic patients?

- ○ Yes
- ○ No
- ○ Other (please specify)

33. If you answered no in the previous question, how did you acquire knowledge on treating adolescent metabolic patients;

- [ ] Fellowships
- [ ] Course
- [ ] Placement in metabolic centre in your country or abroad
- [ ] Non-recognised education programme
- [ ] Other (please specify)

34. If you do not have a separate team for adult metabolic patients in your centre, do you have a specific location “reserved to young adult patients” clinic in your paediatric metabolic clinic?

- ○ Yes
- ○ No
- ○ Other (please specify)

35. Does your hospital provide any additional financial support for transition clinics?

- ◯ Yes
- ◯ No
- ◯ Other (please specify)

36. If you answered yes in the previous question: Could you elaborate on the level and origin of the reimbursement?

37. What are the most difficult challenges your centre/hospital encounter managing the transition process?

- ☐ Lack of time
- ☐ Poor communication between paediatric and adults centres
- ☐ Lack of the reimbursement
- ☐ Lack of interest
- ☐ Lack of knowledge of the medical staff in this area
- ☐ Lack of adult metabolic (physician) positions/vacancies
- ☐ Other (please specify)

38. What would be needed in your hospital for successful transition?

- ☐ More staff specifically dedicated to transition
- ☐ A dedicated coordinator responsible for transition
- ☐ A physical space to have transition clinics
- ☐ Special metabolic training for adult physicians
- ☐ Adult metabolic position
- ☐ Dedicated (or interested) adult physician(s)
- ☐ A transition protocol
- ☐ Other (please specify)

39. Please feel free to add any relevant comments or questions in the following comment box.

We thank you for your kind cooperation and help in filling out this survey!
